# The integrated management of childhood illness (IMCI) and its potential to reduce the misuse of antibiotics

**DOI:** 10.7189/jogh.11.04030

**Published:** 2021-05-22

**Authors:** Susanne Carai, Aigul Kuttumuratova, Larisa Boderscova, Henrik Khachatryan, Ivan Lejnev, Kubanychbek Monolbaev, Sami Uka, Martin W Weber

**Affiliations:** 1 WHO, Regional office for Europe, Copenhagen, Denmark; 2 Witten/Herdecke Universität, Witten, Germany; 3 WHO Country Office, Chisinau, Moldova; 4 WHO Country Office, Yerevan, Armenia; 5 WHO Country Office, Bishkek, Kyrgyzstan; 6 WHO Office Pristina, WHO Regional Office for Europe, Copenhagen, Denmark

## Abstract

**Background:**

The Strategy of the Integrated Management of Childhood Illness (IMCI) was introduced in Central Asia and Europe to address the absence of evidence-based guidelines, the misuse of antibiotics, polypharmacy and over-hospitalization of children. A study carried out in 16 countries analysed the status and strengths of as well as the barriers to IMCI implementation and investigated how different health systems affect the problems IMCI aims to address. Here we present findings in relation to IMCI’s effects on the rational use of drugs, particularly the improved rational use of antibiotics in children, the mechanisms through which these were achieved as well as counteracting system factors.

**Methods:**

220 key informants were interviewed ranging from 5 to 37 per country (median 12). Data was analysed for arising themes and peer-reviewed.

**Results:**

The implementation of IMCI led to improved prescribing patterns immediately after training of health workers according to key informants. IMCI provides standard treatment guidelines and an algorithmic diagnostic- and treatment-decision-tool for consistent decision-making. Doctors reported feeling empowered by the training to counsel parents and address their expectations and desire for invasive treatments and the use of multiple drugs. Improved prescribing patterns were not sustained over time but counteracted by factors such as: doctors prescribing antibiotics to create additional revenues or other benefits; aggressive marketing by pharmaceutical companies; parents pressuring doctors to prescribe antibiotics; and access to drugs without prescriptions.

**Conclusions:**

Future efforts to improve child health outcomes must include: (1) the continued support to improve health worker performance to enable them to adhere to evidence-based treatment guidelines, (2) patient and parent education, (3) improved reimbursement schemes and prescription regulations and their consistent enforcement and (4) the integration of point–of-care tests differentiating between viral and bacterial infection into standards of care. Pre-requisites will be sufficient remuneration of health workers, sound training, improved health literacy among parents, conducive laws and regulations and reimbursement systems with adequate checks and balances to ensure the best possible care.

Child mortality reportedly halved from 12 million to less than 6 million deaths globally during the Millennium Development Goals (MDG) period from 1990 to 2015 [1]. The global strategy of the *Integrated Management of Childhood Illness* (IMCI) launched by the World Health Organization (WHO) and UNICEF in 1995 to end preventable child mortality may be partially credited for this success [2]. The strategy was devised as a three-pronged approach: 1) Improving health worker performance at primary health care (PHC) level, which was subsequently expanded to the referral level, 2) Strengthening health system performance and 3) Enhancing community and family practices.

Next to the improved access to vaccines, the treatment of bacterial infections with antibiotics is likely to have contributed significantly to improved child survival rates [3].

IMCI promotes the availability of antibiotics for children who need them through the adoption of national drugs lists and engaging with Governments to make essential drugs available for children without requiring out-of-pocket payments from parents. IMCI also provides training for health workers on algorithms and guidance based on clinical signs and symptoms on how to differentiate between children who are likely to benefit from antibiotics and those who will not, both at hospital and outpatient levels [4]. Children have several episodes of respiratory tract infections and diarrhoea before their fifth birthday, most of which are self-limiting and not life-threatening. Evidence suggests that in many settings almost three quarters of respiratory tract infections and over 90% of diarrhoeal episodes that are treated with antibiotics would not require antibiotic treatment [5,6]. Over-prescription – in addition to the unnecessary negative effects for the individual patient – is contributing to the emergence of antimicrobial resistance [7-11]. At the same time, underuse of antibiotics persists for the treatment of bloody diarrhoea and pneumonia, which are often bacterial in origin and thus antibiotics would be lifesaving [5,6].

In the European region, IMCI was introduced in the late 1990s in 15 of its member states, namely Albania, Armenia, Azerbaijan, Belarus, Georgia, Kazakhstan, Kyrgyzstan, Moldova, Romania, Russia, Tajikistan, Turkey, Turkmenistan, Ukraine, Uzbekistan and the territory of Kosovo (in accordance with UNSCR 1244, 1999). While huge disparities in childhood mortality existed – and persist – in the region, high mortality was not the main concern in many countries that implemented IMCI. Low quality of care, absence of evidence-based guidelines, misuse of antibiotics and polypharmacy as well as over-hospitalization were the main factors that warranted IMCI implementation [12]. Nearly two decades later, the WHO Regional Office for Europe conducted an in-depth review of IMCI in the WHO European region with the main objective of assessing the status of IMCI implementation, its relevance and effectiveness in providing quality health care to children and to use findings to inform future activities and the renewed IMCI approach.

In this paper, we present findings in relation to implications on the rational use of drugs, particularly the improved rational use of antibiotics for children, the mechanisms through which these were achieved as well as counteracting system factors. Further details and findings are discussed elsewhere [13].

## METHODS

Individual interviews and focus group discussions were carried out by trained WHO staff or consultants at national, district and facility level using semi-structured questionnaires followed by an iterative questioning technique and root cause analysis with key informants in 16 countries and territories, namely Albania, Armenia, Azerbaijan, Belarus, Georgia, Kazakhstan, Kyrgyzstan, Moldova, Romania, Russia, Tajikistan, Turkey, Turkmenistan, Ukraine, Uzbekistan and the territory of Kosovo (in accordance with UNSCR 1244, 1999). This qualitative in-depth approach was chosen to facilitate externalising inherent problems that may be overlooked when familiar routines, forms of interaction, power relationships and established interpretations of situations and strategies are taken for granted.

Key informants involved in or exposed to IMCI implementation were identified through formal and informal networks including through ministries of health (MoH) and WHO country offices. In addition, WHO and UNICEF publications and reports were reviewed for potential key informants and the interviewees were asked to indicate further potential key informants.

A total of 220 key informants were interviewed ranging from 5 to 37 per country with a median of 12. An additional four key informants were interviewed but excluded from further analysis, as their respective countries did not go ahead with IMCI implementation.

Key informants included doctors, nurses and in former Soviet Union countries *feldshers* (health care professional with clinical responsibilities between those of physicians and nurses) providing care to children, representatives of the MoH and country offices of WHO and UNICEF, and other international and non-governmental organizations. Key informants´ profiles can be found in **Table 1**.

**Table 1:** Key informants´ profile

Key informants	No.
Specialists/Doctors working at referral level	56
Doctors working at primary care level	44
Nurses/Feldshers	29
Ministry of health staff	32
Staff of international organizations/NGOs	28
Academia and professional organizations	31
Total	**220**

Investigators were trained in the methodology and interviewing approach and then facilitated open-ended discussions on pre-defined questions and the diagram of the IMCI impact model in search of statements by key informants that could illuminate why IMCI implementation was successful or not in improving child health in the respective country settings. The IMCI impact model can be found in the Figure S1 in the **Online Supplementary Document** and is described more in detail elsewhere [14]. Follow-up questions and an iterative interrogative technique were used to elaborate responses in more detail. A methodology outlining this technique was developed prior to the data collection that is primarily based on *Participatory action research* by Baum et al. [15]. Answers were directly transcribed verbatim or, when consent was granted, audiotaped and then transcribed.

Before the key informant interviews, desk reviews of relevant material were carried out including information collected via a pre-visit-questionnaire completed by the MoH and the WHO country office. The pre-visit-questionnaire as well as the semi-structured questionnaires can be found in the **Online Supplementary Document**. Information collected from the pre-visit-questionnaire, desk review, interviews and focus group discussions was triangulated for cross-validation and analysed for commonly arising themes. Transcripts were reviewed to identify the most frequently mentioned strengths and weaknesses of the IMCI approach. Verbatim comments by key informants specific to each setting were grouped into themes, eg, *IMCI implementation reduced polypharmacy* by the lead investigator.

These groupings were then reviewed by the respective country investigator and a minimum of one other investigator for accuracy and consistency. Final results were agreed upon by consensus of the entire group during a three-day face-to face meeting.

### Patient involvement

The key-informant review was carried out at national, district and health facility level and no individual patient data was included. This study was deemed exempt from ethical review by the WHO Ethics Review Committee ERC.0002743003540/13.

## RESULTS

The IMCI Strategy was introduced through national orientation workshops in 16 countries, of which 14 went ahead with the implementation of the three components. Details on activities and coverage are described elsewhere [13]. In Belarus and Romania no implementation took place and they were hence excluded from the analysis.

### Drug availability

Drugs required to implement the IMCI treatment guidelines were found to have been included in the National Essential Drug lists in 12 of the studied countries. Consistent availability of IMCI drugs free of charge for children was reported by key informants in only four countries (**Table 2**). A list of the IMCI drugs can be found in Table S1 in the **Online Supplementary Document****.**

**Table 2:** Countries reporting the inclusion of IMCI drugs in the National Essential Drug Lists and countries were key informants reported consistent availability of drugs for children

	Reported by # of countries	Albania	Armenia	Azerbaijan	Georgia	Kazakhstan	Kosovo*	Kyrgyzstan	Moldova	Russia	Tajikistan	Turkey	Turkmenistan	Ukraine	Uzbekistan
Inclusion of IMCI drugs in National Essential Drug list	**12**	†	†	†	‡	†	†	†	†	†	†	‡	†	†	†
IMCI drugs are reportedly available free of charge at all times	**4**	‡	‡	‡	‡	†	‡	†	†	‡	‡	‡	†	‡	‡

ICMI – integrated management of childhood illness

*In accordance with the United Nations Security Council resolution 1244 (1999).

†Aspect reported.

‡Aspect not reported.

### Promotion of evidence-based guidelines, rational use of antibiotics and decrease in polypharmacy

Key informants from all countries reported that IMCI implementation promoted the rational use of antibiotics and decreased polypharmacy after training at primary health care and hospital level for different time periods across countries (**Table 3**).

**Table 3:** Reported promotion of rational use of drugs in IMCI-implementing countries in Europe and Central Asia

	Reported by # of countries	Albania	Armenia	Azerbaijan	Georgia	Kazakhstan	Kosovo*	Kyrgyzstan	Moldova	Russia	Tajikistan	Turkey	Turkmenistan	Ukraine	Uzbekistan
IMCI promoted the rational use of antibiotics	**14**	†	†	†	†	†	†	†	†	†	†	†	†	†	†
IMCI decreased polypharmacy by:	**11**	†	†	†	†	†	§	†	†	§	†	§	†	†	†
-Improving prescribing practice through adherence to IMCI algorithm	**10**	§	†	§	†	†	†	†	†	§	†	§	†	†	†
-Addressing parents’ expectation through education and counselling	**11**	†	†	†	§	†	†	†	†	§	†	§	†	†	†

ICMI – integrated management of childhood illness

*In accordance with the United Nations Security Council resolution 1244 (1999).

†Aspect reported.

§No information available.

Two mechanisms were mentioned through which this was achieved.

First, by making available standard treatment guidelines guiding the decision when to prescribe antibiotics, and when not to, based on the classification of severity according to clinical signs and symptoms, such as respiratory rate and chest in-drawing. And second by addressing parent´s expectations through education and counselling. Key informants from most countries reported that there was a strong preference among parents for “medicalized care”, utilizing many drugs as opposed to a single one and preferably by route of injection. There was also the perception that care with newer, more expensive drugs and invasive care is better care. By providing a systematic approach with decision justification, the IMCI algorithm supported doctors in their treatment choices and enabled them to counsel parents better. Key informants from eleven countries reported that IMCI helped to change parent’s perceptions and expectations sufficiently to allow for adherence to evidence-based prescriptions (**Table 3**).

### Aspects influencing antibiotic prescriptions

Key informants in most countries also reported that improved prescribing patterns were not sustained over time and over-prescription of antibiotics re-emerged soon after the training. Factors leading to antibiotic overuse included (1) circumstantial factors such as the accessibility of antibiotics over the counter, (2) demand-side factors such as parents pressuring doctors to prescribe antibiotics and circumventing primary care by accessing secondary specialized care directly (**Box 1**) and (3) supply-side or push factors such as doctors prescribing antibiotics to create additional revenues, being pushed by the pharmaceutical industry or also “just to be on the safe side” (**Box 2**). Key informants reported that primary care was perceived by parents as not “offering any care”.

Box 1 **Key informants’ comments relating to parents´ perceptions and expectation**

There is pressure from parents, they would like rapid recovery of their child, quick treatment solutions and better antibiotics.

[To many parents] quality of care means expensive drugs, IV treatment and IM antibiotics.

The culture of medicine is that you are a good doctor if you prescribe 15 medicines per patient.

Parents want more prescription and go to non- [IMCI] trained specialists. They prescribe more and different drugs.

If you tell parents that you will not prescribe antibiotics for diarrhoea, they will not be happy.

Parents want antibiotics [even] for viral cases as well as parenteral rehydration and if one doctor does not do [as asked], they go to the next.

Parents prefer 3rd generation cephalosporin from Western Europe.

Common belief is that quality of care means expensive drugs, IV treatment, and parenteral antibiotics. Doctors and nurses are given money or gifts by the parents for this “better” treatment.

Box 2 **Key informants’ comments relating to inappropriate antibiotic use**

There are no incentives to use [prescribe] antibiotics rationally but rather how to optimize your income.

There is a problem with pharmaceutical industry and pressure to prescribe certain drugs. Penicillin is too cheap to make a profit.

Pharmaceutical industry providing incentives to doctors prescribing specific drugs. I can tell by type of drugs who was the doctor who treated the patient.

There is a problem with aggressive marketing by pharmaceutical companies – a parent going to pharmacy can be advised to purchase another drug than prescribed by the doctor.

Wide-spread self-prescription and aggressive advertisement of medicines in the media [constitute barriers to IMCI implementation].

IMCI provided systematic knowledge allowing to prescribe the treatment confidently. [However,] over diagnostics and over medicalization is still a problem.

The doctor is coming to the village once a week for Prescription day.

Despite IMCI health workers continue prescribing big number of unnecessary drugs to be on safe side… fear of penalties, administrative rebukes etc.

Still GPs [general practitioners] have a fear and prescribe drugs when not needed.

Doctors would need to know that one is safe when not prescribing antibiotics.

**Table 4** summarizes the number of countries in which key informants reported each of the mentioned aspects.

**Table 4:** Reported aspects influencing prescription of antibiotics in IMCI implementing countries in Europe and Central Asia

	Reported by # of countries	Albania	Armenia	Azerbaijan	Georgia	Kazakhstan	Kosovo*	Kyrgyzstan	Moldova	Russia	Tajikistan	Turkey	Turkmenistan	Ukraine	Uzbekistan
Antibiotics can be purchased over the counter	**13**	**†**	**†**	**†**	**†**	**†**	**†**	**†**	**†**	**†**	**†**	§	**†**	**†**	**†**
Parents pressure doctors for antibiotics	**11**	**†**	**†**	**†**	**†**	**†**	§	**†**	**†**	**†**	§	**†**	**‡**	**†**	**†**
Parents bypass primary care	**12**	§	**†**	§	**†**	**†**	**†**	**†**	**†**	**†**	**†**	**†**	**†**	**†**	**†**
Pharmaceutical industry influences doctors´ decisions	**13**	§	**†**	**†**	**†**	**†**	**†**	**†**	**†**	**†**	**†**	**†**	**†**	**†**	**†**
Doctors prescribe antibiotics to increase revenues	**12**	§	**†**	**†**	**†**	**†**	**†**	**†**	**†**	**†**	**†**	**†**	§	**†**	**†**

ICMI – integrated management of childhood illness

*In accordance with the United Nations Security Council resolution 1244 (1999).

†Aspect reported.

§No information available.

## DISCUSSION

IMCI is considered to have contributed to the reduction of childhood mortality in the implementing countries in the European region in line with findings from other reviews [2,13,16]. Better access to antibiotics for children with bacterial infections is – next to improved access to vaccines (particularly new vaccines, such as anti-pneumococcal vaccines and HiB) – likely to have been one of the key reasons for the successful mortality reduction.

Key informants of our review consistently reported improved prescribing patterns when IMCI trainings were first implemented, but this was not sustained over time. The Cochrane review also could not confirm a consistent effect on prescribing at health facilities or by lay health care workers [2].

Misuse of antibiotics in treating viral upper respiratory infections and watery diarrhoea persists in almost all settings including the countries included in our study [17-19]. Parents´ expectations for quick treatment solutions and a rapid recovery of their child, combined with misconceptions about antibiotics being effective in the treatment of viral infections such as colds and flu raise the demand for antibiotic prescription [20]. Combined with the “just in case attitude” of doctors and the considerations of economic aspects in the provision of care these preconceptions influence health worker’s performance and ability to adhere to guidelines. Often health systems do “no longer desire a healthy child” as one key Informant put it, as the child is required to be labelled sick for creating revenues, both in relation to carrying out diagnostic tests as well as prescribing medication. While IMCI drugs are included in the national drug lists in almost all reviewed countries and supposed to be provided free of charge to children, parents are often required to pay out of pocket for different drugs prescribed, to create revenues or other benefits through incentives of pharmaceutical companies.

Children will continue to have several episodes of respiratory infections and diarrhoea – viral or bacterial - during the first years of life and antibiotics will be the most commonly prescribed therapy among all medications given to children [3,5]. Future efforts to improve health outcomes for children must build on IMCI’s approach to supporting health workers in making evidence-based decisions and confronting parents’ expectations when these are misguided and not in the best interest of the child.

Dialogue in relation to the health systems requirements and needs for reform for successful IMCI implementation were found to be particularly neglected in most countries [13,16]. Provider training and improvement of working conditions, patient and parent education and adequate regulations and their enforcement will go a long way in reducing unnecessary antibiotic use. An important improvement would be access to reliable and affordable point of care tests, which can differentiate between bacterial and viral infections [5,21]. While these tests are not yet and may never be a substitute for clinical appraisal and medical decision making [22], they may be an important factor in the joint patient/parent-doctor decision making process, particularly in settings where primary care offices equipped with not much more than a desk and a pen have little to offer other than a prescription. The global community should enable the development of reliable and affordable point of care tests and negotiate affordable prices for their introduction also in low and middle-income countries. Antibiotic misuse in children must be considered in efforts and action plans to combat antimicrobial resistance [23]. Actions that will be required at global, national, facility and community level in order to sustainably improve the rational use of antibiotics in children are summarized in **Table 5**. 

**Table 5:** Actions required by different levels to improve rational use of antibiotics in children

Global	National health systems	Health care facilities (primary and hospitals)	Communities and homes
Expand evidence base on and raise awareness of overuse of antibiotics in children	Ensure adequate salaries and working conditions	Support and enable health workers to adhere to evidence-based treatment guidelines	Patient and parent education on viral and bacterial infections and harms of misuse of antibiotics for the individual child as well as risk of antimicrobial resistance
Include antibiotic misuse in children in global action plan to combat antimicrobial resistance	Adopt adequate regulations restricting access to antibiotic and ensure their enforcement		
Enable the development and delivery of reliable and affordable point-of-care tests differentiating between bacterial and viral infections in children	Adopt adequate regulations controlling pharmaceutical industries´ access to health workers to stop influence on prescribing decisions	Improve counselling skills for confronting parents´ expectations	Establishment of behavioural norms banning self-medication with antibiotics
Negotiate with manufacturing partners to obtain significant price reductions that facilitate access to these diagnostic tests	Ensure sound pre-service training and on-going medical education	Improving diagnostics: implementation of point of care tests	Establish behavioural norms for allowing children time to recover from self-limiting illness before resorting to antibiotics
	Review financing schemes for potential incentives for misuse of antibiotics		

While antibiotics can be a lifesaving treatment for children with bacterial infections, overuse has become a concern [24,25]. Treatment choices are not always led by evidence-based decisions [26]. Health worker motivation for implementation of IMCI and evidence-based medicine overall is influenced by a variety of incentives, often adverse. The short-term perception of being on the safe side and considerations of personal benefit can outweigh the motivation to avoid long-term problems with antibiotic resistance, inadvertently risking the return to the pre-penicillin era.

### Limitations

A weakness of this review was that it relied on interviews and focus groups with key informants and reviewers, many of which had an intellectual interest in IMCI, as they had been involved in its development and implementation, hence introducing bias. However, end-users of the IMCI algorithms lacking such bias were also interviewed. The need for translation during some key informant interviews and the fact that respondents may not feel comfortable providing answers that present a WHO/UNICEF strategy and/or the country in an unfavourable manner may have contributed to inaccurate answers.

To limit bias and expose inaccurate answers, the iterative questioning technique was used; statements were reviewed together with the respondent for accuracy taking into account forms of interaction, power relationships and established interpretations of the IMCI approach. Care was taken to disentangle policy from what was happening in reality; to differentiate between what was considered to be an effective approach on a theoretical basis and what was observed to have worked well.

Further limitations are mostly due to the qualitative review design; however, it highlights important factors limiting the achievement of IMCI´s full potential and allowed us to explore how systems factors, such as remuneration and regulations affect the problems IMCI is trying to address. Through triangulation of data collected through desk reviews, individual and focus group interviews, findings were cross-validated, and some of the inherent weaknesses in qualitative studies overcome, adding important information to the evidence base.

## CONCLUSION

We call for the continued support to improve and enable health worker performance, patient and parent education and improved regulations and concerted efforts to develop and implement point–of care tests reliably differentiating between viral and bacterial infections in children and to making them available also to low and middle-income countries at affordable price to end misuse of antibiotics in children.

The IMCI strategy has gone a long way in promoting evidence-based medicine and the rational use of drugs. More must be done to end the indiscriminate use of antibiotics and ensure that children get antibiotics when they need them and only then. Future efforts must focus on the sustainability of the rational use of antibiotics in children.

## Additional material

Online Supplementary Document
