# Supplementary material for: The integrated management of childhood illness (IMCI) and its potential to reduce the misuse of antibiotics
Source: J Glob Health. 2021 May 22;11:04030. doi: 10.7189/jogh.11.04030 (PMC8141328; doi:10.7189/jogh.11.04030)
Supplement: Online Supplementary Document [file jogh-11-04030-s001.zip › IMCI review tools/1 Semi structured questionnaire National Level.docx]

Semi-structured questionnaire for key informant interviews

National level

NAME AND CONTACT DETAILS OF THE INTERVIEWED PERSON

___________________________________________________________________________

ORGANIZATION AND FUNCTION OF THE INTERVIEWED PERSON

___________________________________________________________________________

Male_____ Female____

LOCATION: Haceteppe University Social medicine ________________________________________

DATE OF INTERVIEW: ____ ____ / ____ ____ / ____ ____ ____ ____

INTERVIEWED BY: _______________________________________________________________

TIME INTERVIEW BEGAN: ____ ____ : ____ ____

**Note for interviewer**

Please facilitate a discussion about the IMCI impact model and below defined questions in search of statements that could illuminate why IMCI was successful, stalled or reached its maximum potential in addressing child health in the respective country settings. Such statement may for example entail: “ IMCI is too basic for the doctors in country X” or “ All sick children are required to be admitted to the hospital in country Y” etc. When an indication is found that could provide insights into IMCI and a specific country´s conditions please enquire the statement in more detail.

Based on the outcome of the desk review prior to the interview it is recommended to adapt the proposed questionnaire. The questionnaire is conceived as a rough guide – not as a recipe to be followed in detail – it is rather expected the interviewer dives more in detail in some and skips other section based on the type of information and contribution the key informant has to offer.

**Information sheet for key informant**

The interview should take less than an hour. I am kindly asking for your permission if I could go ahead with this interview. All responses will be kept confidential. This means that your interview responses will only be shared with the team members and we will ensure that any information we include in our report does not identify you as the respondent.

Remember, you do not have to talk about anything you do not want to and you may end the interview at any time. Therefore, I sincerely request your cooperation in responding to the following questions. However, at any time during the course of the interview, you are free to terminate the interview.

It is hoped that you will help us understand how child health and services are organized in your country, the barriers you have observed and your ideas.

Outline of the Integrated Management of Childhood Illness (IMCI) impact model
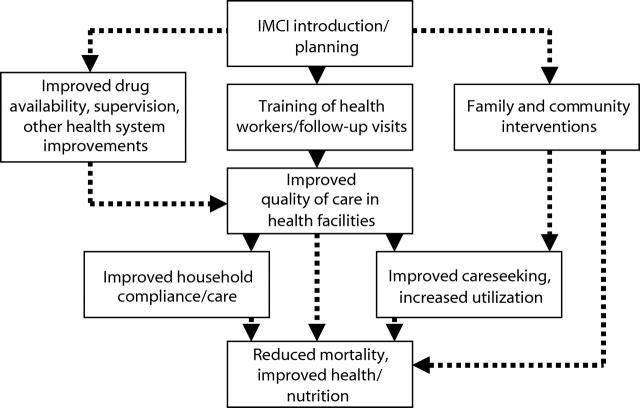


**Source**: Bryce J, Victora CG, Habicht J-P, Vaughan JP, Black RE. The Multi-Country Evaluation of the Integrated Management of Childhood Illness Strategy: Lessons for the Evaluation of Public Health Interventions. American Journal of Public Health. 2004;94(3):406-415.

## Personal information

1. What are your primary responsibilities in this position as it relates to child health and IMCI? How long have you held this position?
2. What is your background? Do you work full time in this capacity? Do you have an additional employment?
3. What previous positions have you been in where you worked on IMCI or other child health strategies?
4. How have you been involved in IMCI implementation? Were yourself trained/oriented in IMCI? If yes, which type of training (11 days, ICATT etc.) and when?

## Child health and IMCI implementation

1. **Do you consider that the IMCI strategy was relevant and useful for your country when it was introduced? Do you think it is still relevant and useful?**
2. There are three components of IMCI strategy: Strengthening System Support, Improving Skills of Health Workers and Community component. What components/activities does IMCI cover in your country? Which of the IMCI components do you consider most useful in your country?
3. How are newborn and child health programme activities coordinated at the national level? Who is responsible for child health, including newborn health? Is there an IMCI coordinator?
4. Is there a National IMCI or child health-working group? What are their functions?
5. **Who is expected to implement/use the algorithmic approach of IMCI?** (Family doctors/General practitioners? Nurses? Community health workers? Who can prescribe drugs/treatment?)
6. Basic IMCI does not teach differential diagnosis skills and the algorithmic approach of IMCI was not primarily designed for use by trained physicians. **Is the IMCI algorithm for primary care relevant for providers caring for children in your country?**
7. **What were the facilitating factors and barriers to implement IMCI in your country?** Was IMCI implemented with sufficient coverage and quality? Were professional association, health care providers opposed to IMCI implementation? Were/are they involved in guideline development? Were/are they involved in supervision? Did you face reluctance to change of health care providers? Did you find them non-confident in skills despite training?
8. **Are there costs for the parents?** **Are IMCI services included in the basic benefit package?** Do you consider informal payments a barrier for parents to access services?
9. **Are drugs and supplies required for IMCI implementation included in the national drug list? Are they always available at the facilities? Are they free of charge to patients (at the PHC/Hospital/both)?** How do you monitor their availability?
10. **Are IMCI protocols aligned to the national protocols?** E.g. management of diarrhoea – stool samples vs. IMCI management of diarrhoea? Other clinical care guidelines?
11. **Is IMCI inline with other National Policies?** For example reporting of diagnosis vs. IMCI classification? Or requirement of referral/ hospitalization?
12. **Is there a regular supervision system for health staff? Specifically in relation to IMCI?** Are there issues related to supervision?
13. **Was there a *Review of health system barriers to implementation of IMCI* in your country? If so, what were the findings and how have they been addressed?**
14. **How much care for sick children is provided by the private sector?** Are there parallel systems in the country providing health care to children? If so, how does this affect the implementation of IMCI? In what ways does MOH work with the private/parallel sector? Were providers from the private/parallel sector included in the training? Are they being supervised? Are they reporting to the MOH?
15. How has IMCI been linked with programming in other areas that are relevant for child health, such as immunization, nutrition?
16. **Are indicators relating to IMCI included in the National Health Information System?**
17. **Do you think that IMCI implementation in your country is sustainable? What are the main facilitating factors and barriers for sustainability of IMCI in your country?**
18. **What is the current support (including financial) to IMCI implementation from the government, from the donors?** Was scaling-up of IMCI strategy ever costed? Tell me about the financing for IMCI and child health more generally?
19. **Has IMCI training been included in undergraduate and/or postgraduate education?** (For different categories of health care providers, which?)
20. **Do you think that implemented IMCI activities have had a significant impact on child health in your country? Please explain.**

## Future of IMCI implementation

1. **What are the most important future actions to improve child health in your country? What do you think should be done in addition or instead of IMCI?** What are in your opinion the most important barriers for children receiving quality care? What would be most helpful to help you improving the care for children? Do you think IMCI has a role to play in the future of your national child health policy?
2. **Is there anything else about IMCI or future child health strategies that we have not discussed that you would like to add?**

*ASK FOR ANY RELEVANT DOCUMENTS AND IF WE CAN FOLLOW UP IF NECESSARY*

*Thank you for participating in this interview. Your responses will help us understand how strategies for treatment of the sick child can best help countries reach child survival & health goals. We thank you for your time.*

TIME INTERVIEW ENDED: ____ ____ : ____ ____
