# Supplementary material for: The integrated management of childhood illness (IMCI) and its potential to reduce the misuse of antibiotics
Source: J Glob Health. 2021 May 22;11:04030. doi: 10.7189/jogh.11.04030 (PMC8141328; doi:10.7189/jogh.11.04030)
Supplement: Online Supplementary Document [file jogh-11-04030-s001.zip › IMCI review tools/2 Focus Group Discussion District level.docx]

Focus Group Discussion

District level

PARTICIPANTS

____________________________________________________________________________________________________________________________________________________________________________________________________________________________________________________________________________________________________________

DATE OF FOCUS GROUP: ____ ____ / ____ ____ / ____ ____ ____ ____

DISCUSSION FACILITATED BY: _________________________________________________________

TIME DISCUSSION BEGAN: ____ ____ : ____ ____

**Note for interviewer**

Please facilitate a discussion about IMCI and below defined questions in search of statements that could illuminate why IMCI was successful, stalled or reached its maximum potential in addressing child health in the respective country settings. Such statement may for example entail: “ IMCI is too basic for the doctors in country X” or “ All sick children are required to be admitted to the hospital in country Y” etc. When an indication is found that could provide insights into IMCI and a specific country´s conditions please enquire the statement in more detail using the **“5 Whys”** technique and/or a **fishbone diagram** (see Annex).

Based on the outcome of the desk review prior to the interview it is recommended to adapt the proposed questionnaire. The questionnaire is conceived as a rough guide – not as a recipe to be followed in detail – it is rather expected the interviewer dives more in detail in some and skips other section based on the type of information and contribution the key informant has to offer.

### Introduction

1. Introduction: Objectives of the IMCI review and the focus group discussion
2. A brief introduction to the rules of focus groups:
3. Everything said and done is confidential and will not be used outside the room except for the purposes of this review;
4. Every statement is right;
5. Please do not hesitate to disagree with someone else; (but do not all talk at once)

### Background/circumstances

1. Ask people to describe who they are and say few words about themselves, including their background, current position, how the position relates to child health and IMCI and how long they have been working in this position, whether they also have an additional job (e.g. private practice or other) and whether they have received training in IMCI? If yes, the type of training (11 days, ICATT etc.) and when? To which extend was IMCI implemented in your district: What proportion of health care providers in your districts were trained in IMCI? What other activities related to IMCI were implemented in your districts? Community component?

### Guiding questions

1. What are the main factors that contributed to success and what are the main constraints you faced while implementing IMCI in your district? Please describe.

Potential areas for prompting, if necessary:

- Shortage/lack of basic equipment/amenities, job aids, drugs: Are drugs and supplies required for IMCI implementation always available at the facilities? How do you monitor their availability?
- Inadequate support from the national level, e.g. policy documents etc.
- Reluctance to change of health care providers/non-confident in skills despite training: Do you regularly supervise health staff? Specifically in relation to IMCI? Are there issues related to supervision?
- Time constraints, referral, parents/caretakers reluctance etc.?

1. Do you think that implemented IMCI activities have had a significant impact on child health in your district? Please explain.
2. What are in your opinion the most important barriers for children receiving quality care in your district? What would be most helpful to help you improving the care for children?

If time permits:

1. Was the IMCI Community component implemented in the communities of your district? What are the most important issues in the communities of your district related to child health?

Ask if they would like to add further comments.

Bring the meeting to a close by summarizing the main points.

*Thank you for participating in this discussion. Your responses will help to understand how strategies for treatment of the sick child can best help countries reach child survival & health goals. We thank you for your time.*

TIME DISCUSSION ENDED: ____ ____ : ____ ____

**Facilitation techniques**

The 5 Whys strategy is an easy and effective tool for uncovering the root of a problem. It's simple and can be applied to almost any problem. Last answer often points to a process: the real root cause should point toward a process that is not working well or does not exist.

Classical answers such as not enough time, not enough investments, or not enough manpower may be true, but are often out of control. Therefore, ask why did the process fail?

Bear in mind, however, that if it does not prompt an intuitive answer, you may need to apply a more comprehensive root cause analysis, e.g. the fishbone diagram

5 Whys

EXAMPLE

Problem statement

During death audit in South Africa the following statement was found in the patient chart of the dead child: “*Oxygen saturation recorded as 66%; no oxygen given; saturation never rechecked” 13 month old Thando with ARI*

1. Why did 13 month old Thando die?

- Because oxygen was not given

1. Why was oxygen not given?

🡪 Because there was no oxygen available on the ward

1. Why was there no oxygen available on the ward?

🡪 Because the oxygen concentrator did not work

1. Why did the oxygen concentrator not work?

🡪 Because it was old and had not been repaired

1. Why had it not been repaired?

🡪 Because there is no maintenance mechanism in place

**FIVE WHYS**

PROBLEM STATEMENT:

________________________________________________________________________________________________________________________________________________________________________________________________________________________________________________________________________________________________________________________________________________________

_____________________________________________________________________

1. Why?

________________________________________________________________________________________________________________________________________________________________________________________________________________________________________________________________________________________________________________________________________________________

_____________________________________________________________________

2. Why?

_______________________________________________________________________________________________________________________________________________________________________________________________________________

_____________________________________________________________________

__________________________________________________________________________________________________________________________________________

3. Why?

_____________________________________________________________________________________________________________________________________________________________________________________________________________

_____________________________________________________________________

__________________________________________________________________________________________________________________________________________

4. Why?

_______________________________________________________________________________________________________________________________________________________________________________________________________________

_____________________________________________________________________

__________________________________________________________________________________________________________________________________________

5.Why? _______________________________________________________________________________________________________________________________________________________________________________________________________________

____________________________________________________________________________________________

____________________________________________________________________________________________

**FISHBONE DIAGRAMM**

´
