# Supplementary material for: The integrated management of childhood illness (IMCI) and its potential to reduce the misuse of antibiotics
Source: J Glob Health. 2021 May 22;11:04030. doi: 10.7189/jogh.11.04030 (PMC8141328; doi:10.7189/jogh.11.04030)
Supplement: Online Supplementary Document [file jogh-11-04030-s001.zip › IMCI review tools/2 Semi structured questionnaire District level..docx]

Semi-structured questionnaire for key informant interviews

**District level**

NAME OF THE INTERVIEWED PERSON

___________________________________________________________________________

ORGANIZATION AND FUNCTION OF THE INTERVIEWED PERSON

___________________________________________________________________________

Male_____ Female____

LOCATION: _________________________________________________________________

____________________________________________________________   Province/Oblast _____________________________________________________________ District/Rayon

DATE OF INTERVIEW: ____ ____ / ____ ____ / ____ ____ ____ ____

INTERVIEWED BY: _______________________________________________________________

TIME INTERVIEW BEGAN: ____ ____ : ____ ____

The interview should take less than an hour. I am kindly asking for your permission if I could go ahead with this interview. All responses will be kept confidential. This means that your interview responses will only be shared with the team members and we will ensure that any information we include in our report does not identify you as the respondent.

Remember, you do not have to talk about anything you do not want to and you may end the interview at any time. Therefore, I sincerely request your cooperation in responding to the following questions. However, at any time during the course of the interview, you are free to terminate the interview.

It is hoped that you will help us understand how child health and services are organized in your country, the barriers you have observed and your ideas.

## Personal information

1. What are your primary responsibilities in this position as it relates to child health and IMCI? How long have you held this position? Do you work full time in this capacity? Do you have an additional employment? Is the salary you are receiving for this position your only/main source of income?
2. What is your background? What training have you received (paediatrics, GP, nursing school etc.?
3. Have you received training in IMCI? If yes, the type of training (11 days, ICATT etc.) and when?
4. Have you been involved in IMCI implementation in your district? If “YES” please describe how.

## Child health and IMCI implementation

1. Who does mainly provide care to children at the first level in your oblast/district (doctors, feldshers, nurses, others)?
2. **To which extend was IMCI implemented in your district**? What proportion of health care providers in your districts were trained in IMCI? What other activities related to IMCI were implemented in your districts? Community component? Other?
3. **What are the main factors that contributed to success and what are the main constraints you faced while implementing IMCI in your district?** Please describe.
4. **Are drugs and supplies required for IMCI implementation always available at the facilities in your district? Are they free of charge for caretakers (at the PHC/Hospital/both)??** How do you monitor their availability?
5. **Do you receive the necessary policy support for IMCI implementation from the national level?** Is IMCI included in the Basic Benefit Package? Are IMCI protocols aligned to the national protocols, e.g. management of diarrhoea – stool samples vs. IMCI management of diarrhoea? Is IMCI inline with other National Policies? Reporting of diagnosis vs. IMCI classification? Requirement of referral, hospitalization?
6. **Do you face staff shortages and/or high staff turn-over in your district? Has this impacted the implementation of IMCI?**
7. **Did you face reluctance to change/apply IMCI of health care providers? If so, why?**

Basic IMCI does not teach differential diagnosis skills and the algorithmic approach of IMCI was not primarily designed for trained physicians. Has this created any problems?

1. **Do you regularly supervise health staff? Specifically in relation to IMCI?** Are there issues related to supervision?
2. **Have there been any changes in the referral system after the introduction of IMCI in your district?** Do you think that the number of children who are referred to the hospital has changed after introduction of IMCI? What are the challenges with referral in your experience?
3. **Did you face reluctance of parents/caretakers when implementing IMCI?** If so, why? Rational drug use not inline with parent´s perception of good quality care
4. Was the IMCI Community component implemented in the communities of your district? What are the most important issues in the communities of your district related to child health?
5. **Do you think that implemented IMCI activities have had a significant impact on child health in your district?** Please explain.

## Future of IMCI implementation

1. **What are the most important future actions to improve child health in your district?** What would be most helpful to help you improving the care for children? What are in your opinion the most important barriers for children receiving quality care in your district?
2. **Is there anything else about IMCI or future child health strategies that we have not discussed that you would like to add?**

*ASK FOR ANY RELEVANT DOCUMENTS AND IF WE CAN FOLLOW UP IF NECESSARY*

*Thank you for participating in this interview. Your responses will help us understand how strategies for treatment of the sick child can best help countries reach child survival & health goals. We thank you for your time.*

TIME INTERVIEW ENDED: ____ ____: ____ ____
