# Supplementary material for: The integrated management of childhood illness (IMCI) and its potential to reduce the misuse of antibiotics
Source: J Glob Health. 2021 May 22;11:04030. doi: 10.7189/jogh.11.04030 (PMC8141328; doi:10.7189/jogh.11.04030)
Supplement: Online Supplementary Document [file jogh-11-04030-s001.zip › IMCI review tools/3 Focus Group Discussion First level.docx]

Focus Group Discussion

Facility level

PARTICIPANTS

_________________________________________________________________________________________________________________________________________________________________________________________________________________________________

___________________________________________________________________________

DATE OF FOCUS GROUP : ____ ____ / ____ ____ / ____ ____ ____ ____

DISCUSSION FACILITATED BY: _________________________________________________________

TIME DISCUSSION BEGAN: ____ ____ : ____ ____

**Note for interviewer**

Please facilitate a discussion about IMCI and below defined questions in search of statements that could illuminate why IMCI was successful, stalled or reached its maximum potential in addressing child health in the respective country settings. Such statement may for example entail: “ IMCI is too basic for the doctors in country X” or “ All sick children are required to be admitted to the hospital in country Y” etc. When an indication is found that could provide insights into IMCI and a specific country´s conditions please enquire the statement in more detail using the **“5 Whys”** technique and/or a **fishbone diagram** (see Annex).

Based on the outcome of the desk review prior to the interview it is recommended to adapt the proposed questionnaire. The questionnaire is conceived as a rough guide – not as a recipe to be followed in detail – it is rather expected the interviewer dives more in detail in some and skips other section based on the type of information and contribution the key informant has to offer.

### Introduction

1. Introduction: Objectives of the IMCI review and the focus group discussion
2. A brief introduction to the rules of focus groups:
3. Everything said and done is confidential and will not be used outside the room except for the purposes of this review;
4. Every statement is right;
5. Please do not hesitate to disagree with someone else; (but do not all talk at once)

### Background/circumstances

1. Ask people to describe who they are and say few words about themselves, including their background, current position, how long they have been working in this position, whether they also have an additional job (e.g. private practice or other) and whether they have received training in IMCI? If yes, the type of training (11 days, ICATT etc.) and when? How many children are seen at your facility each day on average (low season/peak season)?

Which type of examination/diagnostic tests do you carry out at your facility?

### Guiding questions

1. How useful did you find the IMCI training? Are you using the IMCI chart/algorithm during your daily work? In your opinion, how relevant is IMCI for your work? Does it address your needs? Has IMCI helped or hindered your work?

Potential areas for prompting, if necessary:

- Which proportion of children under 5 presents with symptoms, which are not covered in the IMCI algorithm? How do you assess and treat children above 5 years of age?
- Basic IMCI does not teach differential diagnosis skills and the algorithmic approach of IMCI was designed primarily for use by community health workers, not trained physicians.
- How do you report on the number of children seen at your facility? Specifically are IMCI classifications incorporated in the reporting systems or do you report by diagnosis?
- How many of the children do you refer? What are the challenges with referral in your experience?

1. Which factors helped you to accept and use new practices and procedures included in IMCI guidelines and which prevented you? Please describe.

Potential areas for prompting, if necessary:

- Shortage/lack of basic equipment/amenities, job aids, drugs: Are drugs and supplies required for IMCI implementation always available at your facility? How often do parents/caregivers have to buy them outside the facility? How often do you prescribe drugs other than drugs covered in the IMCI algorithm?
- Reluctance to change/non-confident in skills despite training: Are you regularly supervised? Specifically in relation to IMCI? Are there issues related to supervision? Please explain whether and how supervision helps/limits you to improve service quality?
- Time constraints, referral, parents/caretakers reluctance etc.?

1. What are in your opinion the most important barriers for children receiving quality care? What would be most helpful to help you improving the care for children?

If time permits:

1. Was the IMCI Community component implemented in the community of your health facility? What are the most important issues in your community related to child health?

Ask if they would like to add further comments.

Bring the meeting to a close by summarizing the main points.

*Thank you for participating in this discussion. Your responses will help to understand how strategies for treatment of the sick child can best help countries reach child survival & health goals. We thank you for your time.*

TIME DISCUSSION ENDED: ____ ____ : ____ ____

**Facilitation techniques**

The 5 Whys strategy is an easy and effective tool for uncovering the root of a problem. It's simple and can be applied to almost any problem. Last answer often points to a process: the real root cause should point toward a process that is not working well or does not exist.

Classical answers such as not enough time, not enough investments, or not enough manpower may be true, but are often out of control. Therefore, ask why did the process fail?

Bear in mind, however, that if it does not prompt an intuitive answer, you may need to apply a more comprehensive root cause analysis, e.g. the fishbone diagram

5 Whys

EXAMPLE

Problem statement

During death audit in South Africa the following statement was found in the patient chart of the dead child: “*Oxygen saturation recorded as 66%; no oxygen given; saturation never rechecked” 13 month old Thando with ARI*

1. Why did 13 month old Thando die?

- Because oxygen was not given

1. Why was oxygen not given?

🡪 Because there was no oxygen available on the ward

1. Why was there no oxygen available on the ward?

🡪 Because the oxygen concentrator did not work

1. Why did the oxygen concentrator not work?

🡪 Because it was old and had not been repaired

1. Why had it not been repaired?

🡪 Because there is no maintenance mechanism in place

**FIVE WHYS**

PROBLEM STATEMENT:

________________________________________________________________________________________________________________________________________________________________________________________________________________________________________________________________________________________________________________________________________________________

_____________________________________________________________________

1. Why?

________________________________________________________________________________________________________________________________________________________________________________________________________________________________________________________________________________________________________________________________________________________

_____________________________________________________________________

2. Why?

_______________________________________________________________________________________________________________________________________________________________________________________________________________

_____________________________________________________________________

__________________________________________________________________________________________________________________________________________

3. Why?

_____________________________________________________________________________________________________________________________________________________________________________________________________________

_____________________________________________________________________

__________________________________________________________________________________________________________________________________________

4. Why?

_______________________________________________________________________________________________________________________________________________________________________________________________________________

_____________________________________________________________________

__________________________________________________________________________________________________________________________________________

5.Why? _______________________________________________________________________________________________________________________________________________________________________________________________________________

____________________________________________________________________________________________

____________________________________________________________________________________________

**FISHBONE DIAGRAMM**

´
