# Supplementary material for: The integrated management of childhood illness (IMCI) and its potential to reduce the misuse of antibiotics
Source: J Glob Health. 2021 May 22;11:04030. doi: 10.7189/jogh.11.04030 (PMC8141328; doi:10.7189/jogh.11.04030)
Supplement: Online Supplementary Document [file jogh-11-04030-s001.zip › IMCI review tools/3 Semi structured questionnaire First level.docx]

Semi-structured questionnaire for key informant interviews

Facility level

NAME OF THE INTERVIEWED PERSON

___________________________________________________________________________

ORGANIZATION AND FUNCTION OF THE INTERVIEWED PERSON

___________________________________________________________________________

Male_____ Female____

LOCATION: _________________________________________________________________

____________________________________________________________   Province/Oblast _____________________________________________________________ District/Rayon _____________________________________________________________ Community

DATE OF INTERVIEW: ____ ____ / ____ ____ / ____ ____ ____ ____

INTERVIEWED BY: _______________________________________________________________

TIME INTERVIEW BEGAN: ____ ____ : ____ ____

The interview should take less than an hour. I am kindly asking for your permission if I could go ahead with this interview. All responses will be kept confidential. This means that your interview responses will only be shared with the team members and we will ensure that any information we include in our report does not identify you as the respondent.

Remember, you do not have to talk about anything you do not want to and you may end the interview at any time. Therefore, I sincerely request your cooperation in responding to the following questions. However, at any time during the course of the interview, you are free to terminate the interview.

It is hoped that you will help us understand how child health and services are organized in your country, the barriers you have observed and your ideas.

## Personal information

1. What are your primary responsibilities in this position as it relates to child health? How long have you held this position?
2. What is your background? What training have you received (paediatrics, GP, nursing school etc.?
3. Do you work full time in this capacity? Do you treat children also in private practice? Do you have an additional employment? Is the salary you are receiving for this position your only/main source of income?

## Child health and IMCI implementation

1. How many children under 5 are seen at your facility each day on average (low season/peak season)? How many of them do you see (manage) personally?
2. What type of IMCI training did you receive (11 days, ICATT) and when? Which other in-service trainings have you received (TB, HIV, data management, other) and when?
3. How useful did you find the IMCI training for your daily work? Please provide details. Are you using the IMCI charts/algorithms during your daily work?
4. In your opinion, has IMCI helped or hindered your work? Which proportion of children under 5 presents with symptoms, which are not covered in the IMCI algorithm? Which type of examination/diagnostic tests do you carry out at your facility that are not covered in the IMCI algorithm? How do you assess and treat children above 5 years of age?
5. Basic IMCI does not teach differential diagnosis skills and the algorithmic approach of IMCI was not primarily designed for trained physicians. Has this created any problems for you or your colleagues?
6. How do you record and report about children under 5 who have been assessed and managed? Specifically are IMCI classifications incorporated in the reporting systems or do you report by diagnosis?
7. What helped you to accept and use new practices and procedures included in IMCI guidelines and what prevented you? Please describe.
8. Are you regularly supervised? Specifically, in relation to IMCI? Are there issues related to supervision?
9. Are drugs and supplies required for IMCI implementation always available at your facility? Describe in details if there are any problems? How often do parents/caregivers have to buy them outside the facility? How often do you prescribe drugs other than drugs covered in the IMCI algorithm?
10. Have there been any changes in the referral system after the introduction of IMCI at your health facility? Do you think that the number of children who are referred to the hospital has changed after introduction of IMCI at your health facility? What are the challenges with referral in your experience?
11. In your experience, how do caretakers of the children under 5 react to the use of IMCI guidelines and approaches? Please provide details
12. What are the most important issues in your community related to child health?

Was the IMCI Community component implemented in the community of your health facility? If yes, please describe. Do you have any health promotion activities in your health facility that support IMCI implementation? If yes, please describe.

## Future of IMCI implementation

1. What would be most helpful to help you improving the care for children in your facility?

1. Is there anything else about IMCI or future child health strategies that we have not discussed that you would like to add?

TIME INTERVIEW ENDED: ____ ____ : ____ ____
