# Supplementary material for: The integrated management of childhood illness (IMCI) and its potential to reduce the misuse of antibiotics
Source: J Glob Health. 2021 May 22;11:04030. doi: 10.7189/jogh.11.04030 (PMC8141328; doi:10.7189/jogh.11.04030)
Supplement: Online Supplementary Document [file jogh-11-04030-s001.zip › IMCI review tools/Guide to question 4 of pre-visit questionnaire .docx]

# **IMCI Country background information**

Date completed:

**Country info**

Name:

Country population

Population Under 5

Under 5-mortality rate (*rate/year*)

Population Under 1

IMR (*rate/year*)

Total number of health workers (*number/year*)

Number of health workers who provide care to children Under 5 (*number/year*)

## **IMCI introduction**

INTRODUCTION

1. When was IMCI introduced in the country? (*year*)

Main reason for IMCI introduction (*high mortality rate, need to improve child health, donors pressure, others*)

How IMCI was introduced? (*National meeting, model training, others*)

1. Are there policy documents supporting the introduction of IMCI (*yes/no*)
2. Have the essential IMCI drugs been included into the National Essential Drug list? (*yes/no*)

If “YES” indicate the year

1. In your opinion what were the strengths and weaknesses of the IMCI introduction in your country (*Please indicate at least three strong and three weak points*)

ADAPTATION

1. When have the IMCI guidelines been adapted? (*year*)
2. Was external support provided for the national adaptation (*yes/no*)

If “YES” please describe (*high/medium/low*)

1. Has the academic community been involved into the process of IMCI adaptation? (yes/no)

If “YES”, please provide details

1. Were representatives of first level health facilities involved in the IMCI adaptation? (*yes/no*)
2. Has the management of newborn problems (*less than 2 weeks of age*) been included into the national adaptation of IMCI guidelines (*yes/no*)

If “YES”, indicate the year

1. Were there any subsequent IMCI adaptations? (*yes/no*)

If “YES” indicate when and what was changed (*if several adaptations took place describe all of them*)

## **IMCI implementation**

HEALTH SYSTEM SUPPORT

1. Is there an IMCI coordinator at the national level? (*yes/no*)

If “YES”, please indicate name and contact details of the current coordinator

1. Are there persons responsible for the IMCI implementation at the peripheral level? (yes/no)

If “YES”, please provide details (level and title) and scope of responsibilities

If “YES”, please provide details on how and how often IMCI implementation is being supervised, how many (or approximate percentage) of the supervisors received IMCI training themselves; how are the results of supervision used.

1. Did nationwide IMCI implementation take place (*yes/no*)

If “YES”, please indicate year

If “NO”, please indicate what approximate proportion of Under 5 population is covered by IMCI impel

1. Was IMCI implemented in both urban and rural areas? *(yes/no*)

If “NO”, please, provide details

1. Was IMCI included into ongoing supervisory activities (yes/no)
2. Has any periodic review of IMCI implementation took place in the country? (*yes/no*)

If “YES”, please indicate the year(s) and what were the major findings

1. Has any type of IMCI-related research been conducted in the country? (*yes/no*)

If “YES”, please provide details

1. Are IMCI drugs and essential equipment always available for the first level health facilities (*yes/no*)

If “NO”, please provide details of the major problems

1. Have IMCI classifications been incorporated into the National Health Information System? (*yes/no*)

If “NO”, please indicate the reasons

TRAINING/CAPACITY BUILDING

1. When did the first IMCI training take place (*year*)
2. How many IMCI trainers do currently exist in the country

If no exact number is available, please provide an estimate

1. Is there (are there) a national/subnational IMCI training centre(s) (*yes/no*)

If “YES” indicate the year when it (they) was (were) established and provide details on the activities

1. How many eligible health workers (*those who take care of children Under 5*) have received formal IMCI training (*of any type and duration*) to date (*indicate total number or approximate percentage of eligible health workers trained*)
2. Who provides major financial support to the regular IMCI training activities (*government, local donors, international organizations, others*)
3. Please indicate what types of IMCI training are used in the country (*formal training courses, e-learning, self-learning options, others*), please indicate respective duration

If different types of IMCI training were introduced in the country, please indicate approximately what percentage of health workers received which type of training.

1. Have IMCI guidelines been introduced into preservice training of health workers (yes/no)?

If “YES”, please indicate year when IMCI was incorporated into preservice training, and specify in which types of training programmes IMCI guidelines were included (*for medical doctors, nurses, others and if possible, indicate the number of academic hours allocated to IMCI teaching*)

1. Did the country adapt and introduce the WHO Pocket Book of Hospital Care for children?

If so, please indicate the year and activities to introduce the Pocket Book to health workers (*orientation seminars, distribution of Pocket Books to health facilities, formal training, others*)

1. Have special activities to strengthen IMCI community component been implemented in the country (*yes/no*)

If “YES”, please, indicate the year when activities started and briefly describe those activities

STRENGTHS AND WEAKENESSES

1. In your opinion what were the strengths and weaknesses of IMCI implementation in the country (*please indicate at least three strong and three weak points*)

# **IMCI future**

1. Are there any officially adopted plans for continuation of IMCI in the nearest future? (*yes/no*)

If “YES”, please provide details

1. Are there any officially adopted plans to improve child health in the country in the nearest future? (*yes/no*)

If “YES”, please provide details
