# Supplementary material for: The integrated management of childhood illness (IMCI) and its potential to reduce the misuse of antibiotics
Source: J Glob Health. 2021 May 22;11:04030. doi: 10.7189/jogh.11.04030 (PMC8141328; doi:10.7189/jogh.11.04030)
Supplement: Online Supplementary Document [file jogh-11-04030-s001.zip › IMCI review tools/Pre-visit questionnaire.docx]

Pre-visit Questionnaire

1. Please list and share available background information^[[1]](#footnote-1)^ in relation to IMCI in your country? ___________________________________________________________________________________________________________________________________________________________________________________________________________________________________________________________________________________________________________________________________________________________________________________________________________________________________________________________________________________________________

Please share these documents or provide contact details of a person who would be able to.

1. Please indicate relevant stakeholders^[[2]](#footnote-2)^ we may contact for
   1. Key informant interviews
   2. Focus group discussion

_______________________________________________________________________________________________________________________________________________________________________________________________________________

_______________________________________________________________________________________________________________________________________________________________________________________________________________

__________________________________________________________________________________________________________________________________________

1. Please indicate a district/first level health facility where children are cared for (using IMCI or not) suitable for a field visit

__________________________________________________________________________________________________________________________________________

_______________________________________________________________________________________________________________________________________________________________________________________________________________

1. Please provide a short summary on IMCI implementation in your country^[[3]](#footnote-3)^
   1. Decision on and start of implementation of IMCI (When? Who? What? Why? Where?)
   2. Please share who was/is supposed to use IMCI for child management, e.g. doctors, nurses, community health workers etc.? Were supervision mechanisms put in place?
   3. Important milestones (IMCI policy/action plan, adopted IMCI national guidelines, IMCI pre-service training, etc.)
   4. State as of now
   5. Strength and weaknesses
   6. Recommendations for improving child health in your country (with or without IMCI)

If IMCI was not implemented, please state why not. _____________________________________________________________________

_________________________________________________________________________________________________________________________________________________________________________________________________________________________________________________________________________________________________________________________________________________________

_____________________________________________________________________

_________________________________________________________________________________________________________________________________________________________________________________________________________________________________________________________________________________________________________________________________________________________

_________________________________________________________________________________________________________________________________________________________________________________________________________________________________________________________________________________________________________________________________________________________

_____________________________________________________________________

_________________________________________________________________________________________________________________________________________________________________________________________________________________________________________________________________________________________________________________________________________________________

_____________________________________________________________________

_________________________________________________________________________________________________________________________________________________________________________________________________________________________________________________________________________________________________________________________________________________________

_____________________________________________________________________

1. Such as technical reports, notes from IMCI introduction workshop, IMCI health facility surveys, rapid assessment/monitoring reports of IMCI implementation at facility level, reports on the implementation of the IMCI community component, implementation of pre-service training etc. and any other documents you deem useful for the purpose of the IMCI review in the European region [↑](#footnote-ref-1)
2. Such as stakeholders from MoH, Academia/ University, International organizations, Health facility managers, Health workers, Community level implementers, other [↑](#footnote-ref-2)
3. If you whish you may use the attached questionnaire for helping you with the summary [↑](#footnote-ref-3)
