# Supplementary material for: The integrated management of childhood illness (IMCI) and its potential to reduce the misuse of antibiotics
Source: J Glob Health. 2021 May 22;11:04030. doi: 10.7189/jogh.11.04030 (PMC8141328; doi:10.7189/jogh.11.04030)
Supplement: Online Supplementary Document [file jogh-11-04030-s001.zip › Online Supplementary Document .pdf]

Online Supplementary Document

Figure S1 - IMPACT MODEL IMCI

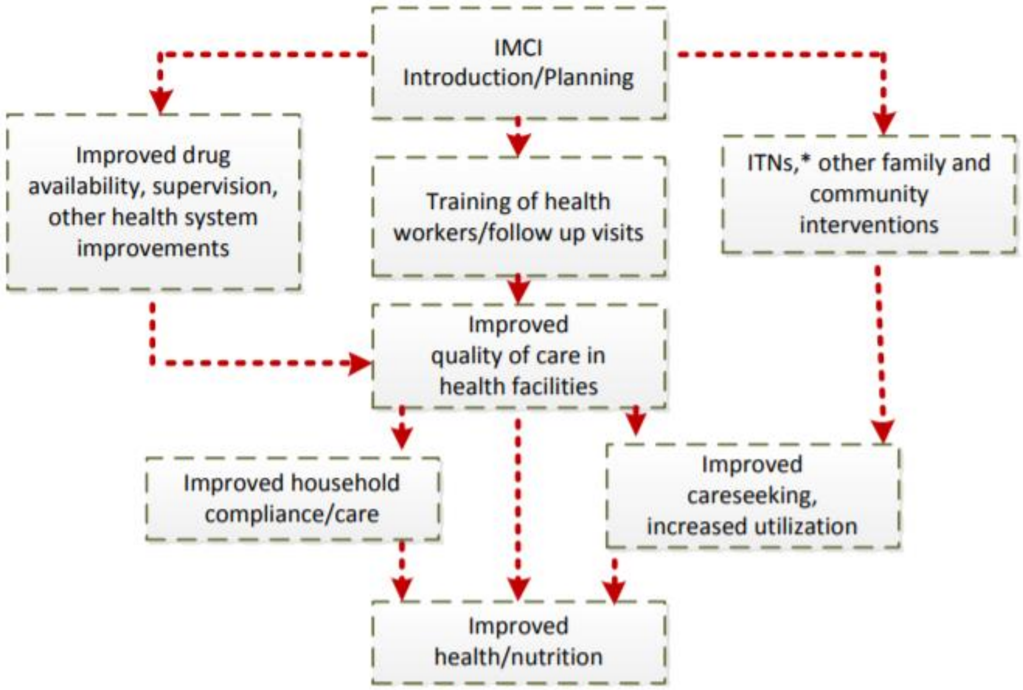

Source: The multi-country evaluation of IMCI effectiveness, cost and impact (MCE). Progress report May 2001–April 2002. Geneva: World Health Organization; 2002 (WHO/FCH/CAH/02.16; http://apps.who.int/iris/handle/10665/67381).
*INTs – Insecticide-Treated Mosquito Nets.

Table S1. LIST OF ESSENTIAL IMCI RECOMMENDED DRUGS

DRUG
Cotrimoxazole<br>— Adult tablet (80 mg trimethoprim + 400 mg sulphamethoxazole)<br>— Paediatric tablet (20 mg trimethoprim + 100 mg sulphamethoxazole)<br>— Syrup (40 mg trimethoprim + 200 mg sulphamethoxazole)
Amoxycillin<br>— Tablet (250 mg)<br>— Syrup (125 mg per 5 ml)
Chloramphenicol Intramuscular (1000 mg vial)
Gentamicin Intramuscular<br>— (2 ml vial containing 20 mg) OR<br>— (2 ml vial containing 80 mg)
Benzylpenicillin (600 mg vial [1 000 000 units]
ORS 500ml sachet
Hartmanns solution 500ml bottles
Nalidixic acid tablets (250mg)
Metronidazole (200mg tabs)
Erythromycin Tablets (250 mg)
Mebendazole 500 mg tabs
Iron 200mg
Folic Acid tabs (5mg)
Paracetamol<br>— Tablet (500 mg)<br>— Tablet (100 mg)
Gentian Violet
Mycostatin (20ml)
Diazepam – vials
10% glucose ½ litre bottle
Salbutamol (tabs) 2mg
Salbutamol inhaler
Salbutamol nebulization solution (50ml bottle)
Vitamin A (capsules) soft gelatmous 100,000 and 200,000 units
Vaccines
Tetracycline Tablets (250 mg) and tetracycline eye ointment

S3. TOOLS: QUESTIONNAIRES USED AT FIRST, DISTRICT AND NATIONAL LEVEL FOR INDIVIDUAL INTERVIEWS AND FOCUS GROUP DISCUSSION AND THE PRE-VISIT QUESTIONNAIRE *

- 1 Focus Group Discussion National level.docx
- 1 Semi structured questionnaire National Level.docx
- 2 Focus Group Discussion District level.docx
- 2 Semi structured questionnaire District level..docx
- 3 Focus Group Discussion First level.docx
- 3 Semi structured questionnaire First level.docx
- Guide to question 4 of pre-visit questionnaire .docx
- Pre-visit questionnaire.docx

*see attached Zip file
